# Relation between Self-Organization and Wear Mechanisms of Diamond Films

**DOI:** 10.3390/e20040279

**Published:** 2018-04-13

**Authors:** Vitali Podgursky, Andrei Bogatov, Maxim Yashin, Sergey Sobolev, Iosif S. Gershman

**Affiliations:** 1 Department of Mechanical and Industrial Engineering, Tallinn University of Technology, Ehitajate tee 5, 19086 Tallinn, Estonia; 2 Department of Mechanical Engineering, Gubkin Russian State University of Oil and Gas, Leninsky Prospect 65, 119991 Moscow, Russia; 3 Joint Stock Company Railway Research Institute, Moscow State Technological University “Stankin” (MSTU “STANKIN”), 3rd Mytischinskaya Street 10, 129851 Moscow, Russia

**Keywords:** self-organization, tribology, diamond films

## Abstract

The study deals with tribological properties of diamond films that were tested under reciprocal sliding conditions against Si_3_N_4_ balls. Adhesive and abrasive wear are explained in terms of nonequilibrium thermodynamic model of friction and wear. Surface roughness alteration and film deformation induce instabilities in the tribological system, therefore self-organization can occur. Instabilities can lead to an increase of the real contact area between the ball and film, resulting in the seizure between the sliding counterparts (degenerative case of self-organization). However, the material cannot withstand the stress and collapses due to high friction forces, thus this regime of sliding corresponds to the adhesive wear. In contrast, a decrease of the real contact area leads to the decrease of the coefficient of friction (constructive self-organization). However, it results in a contact pressure increase on the top of asperities within the contact zone, followed by material collapse, i.e., abrasive wear. Mentioned wear mechanisms should be distinguished from the self-lubricating properties of diamond due to the formation of a carbonaceous layer.

## 1. Introduction

Friction and wear mechanisms were intensively investigated in the past. Three laws of friction state that a friction force (*F*) depends linearly on the normal load (*W*) and does not depend on the nominal area of contact and velocity (*V*). These laws can be also formulated as dynamical coefficient of friction (COF) independence of applied normal load, nominal area of contact, and velocity [1].

The real contact area (*A*) is a key parameter for tribological characterization. The real contact area is only a fraction of the nominal contact area, since contacts occur only on top of asperities. The linearity of Amontons friction law *F* = *μW* (*μ*—coefficient of friction) is a consequence of the fact that the real contact area is almost directly proportional to the applied normal load. The linear dependence of the real contact area on the small normal load was shown theoretically by Greenwood and Williamson [2] for Gaussian and by Bush et al. [3,4] for randomly rough surfaces, respectively. It was suggested by Bowden and Tabor [5] that *F* = *τA*, where *τ* is shear strength due to adhesion on the interface. In practice, friction is nonlinear in nature. For instance, it was shown that the COF can depend on the sliding velocity, load, and contact area [1,6,7,8,9,10].

By definition, the adhesive and abrasive wear are related to the concept of the real contact area. The larger real contact area between the sample and counterbody (other things being equal) leads to stronger adhesion between bodies, thus to the stronger adhesive wear. Hardness is the important material property for the understanding of the abrasive wear, it is characteristic of material to withstand the stress without plastic deformation. Material with the higher hardness experiences lower abrasive wear. However, the amount of stress in the material body depends on the real contact area and the applied load.

The nonlinear character of friction can be described by nonequilibrium thermodynamics [11,12]. Theory predicts conditions that can lead to self-organization, which can reduce wear. The definition and physical meaning of self-organization relate to the formation of dissipative structures [11,12,13]. These are stationary processes leading to the negative excessive entropy production [11,14,15]. Elastodynamic, thermoelastodynamic, etc. instabilities can trigger the formation of dissipative structures [12,13]. Wear is one of the many processes occurring during sliding. The energy that is induced by friction can be consumed by other processes, thus reducing the wear rate [14]. The entropy production (∂*S*/∂*t*), i.e., a rate of change in entropy due to processes that are occurring within the tribological system, can be written as follows:

(1) ∂S/∂t=JX,

where thermodynamic flow *J* = *μWV*, thermodynamic force *X* = *μWV*/(*λT*^2^*A*), entropy (*S*), time (*t*), temperature (*T*), and heat conductivity (*λ*) [11,15]. It is assumed that the work by friction force *F* = *μW* is *μWV* per unit time, which dissipates within the thin contact layer. The derivation of thermodynamic flow and force for frictional sliding can be found elsewhere [15,16].

The stability conditions for the thermodynamic system can be analyzed using the Lapunov’s function *δ*^2^*S* (where *δ*^2^*S* is the second variation of entropy). Self-organization can occur in the system if inequality ∂/2∂*t* (*δ*^2^*S*) ≥ 0 is not fulfilled. The next equation shows how the calculation of this derivative can be simplified [11,16]:

(2) $$\frac{\partial }{2\partial t}\left(\delta^{2}S\right)=\frac{1}{2}\delta^{2}\left(\frac{{\left(\mu WV\right)}^{2}}{\lambda T^{2}A}\right)=\delta X\delta J=\delta \left(\mu WV\right)\delta \left(\frac{\mu WV}{\lambda T^{2}A}\right)\geq 0.$$

In other words, only variations of thermodynamic flow and force must be evaluated in order to estimate the variation of entropy. 

Wear of diamond is a velocity- and load-dependent process [9,10]. In the case of sliding in the air, the chemical passivation of dangling carbon bonds by species from ambient environment decreases COF [17,18,19,20]. Tribological behavior of diamond films during running-in is affected by the initial roughness of contacting bodies. Higher surface roughness results in higher initial friction with a following decrease of COF value due to surface smoothing [21,22,23]. In addition, stress-induced mechano-chemical formation of the carbonaceous lubricating layer takes places already at the beginning of running-in, leading to a decrease of COF value as well [21,22,23,24,25,26,27]. In spite of the counterbodies smoothing and formation of carbonaceous layer, the different types of COF curves were observed after the tests on diamond films. An increase of the COF value at the beginning of sliding was found in the tests with a larger normal load on the thicker films [28,29] and on the thinner films with the same normal load [30], which indicates the importance of stress and deformation to explain the tribological behavior of diamond films. 

The present review is based on the analysis of deflection phenomenon on diamond films investigated in our recent studies [31,32,33,34,35]. The deflection is considered as a plastic deformation of the whole film within contact zone and it should be distinguished from the plastic deformation of the asperities [36]. Diamond is a brittle material, thus the fracture of asperities can occur [1]. The origin of deflection phenomenon can be the film/substrate structure and film bending (elastic and plastic deformation) during sliding [31,32]. The film deflection increases with an increasing of the test duration and normal load and can be explained in terms of fatigue [31].

The study aims to estimate the variation of the real contact area with the film bending and surface roughness alteration and interpret tribological behavior of diamond films by means of self-organization.

## 2. Materials and Methods 

The tribological system that is under investigation can be viewed as a thin hard film (diamond film) on a softer substrate (Si, WC-Co). Two types of diamond films (nanocrystalline diamond (NCD), microcrystalline diamond (MCD)) were investigated, see for details our publications [26,31,32,33,34,35,37,38]. The NCD films were grown under different conditions, therefore they are abbreviated by NCD-1 [31,32,37], NCD-2 [26,38] and NCD-3 [33]. The thickness of the NCD films was as follows: 4.8, 9 and 22 µm (NCD-1) and 0.8 µm (NCD-2, NCD-3). The thickness of MCD films was 5 µm. The films were characterized by Raman spectroscopy, optical microscopy, atomic force microscopy (AFM), scanning electron microscopy (SEM), and mechanical profilometry. The reciprocal sliding tests [26,39] were carried out at room temperature in ambient air using Si_3_N_4_ balls. The frequency varied between 2–10 Hz and normal load between 0.5–3 N. ISO 3D parameter Surface Area Ratio (Sdr) was used in the current research to characterize the contact area between the ball and the film. Sdr is defined as the increment of the total (nominal) surface area relative to the sampling area in the *XY* (surface) plane.

## 3. Theoretical Background

The real contact area between the counterparts changes during sliding due to the variation of the local roughness (wear of material), deformation of the contacting surfaces, etc. In general, the influence of each of the mentioned factors on the real contact area can be described by introducing parameters *ψ*_1_, *ψ*_2_, etc. [11]. In the present study, for the sake of simplicity, parameter *ψ* corresponds to either the diamond film deformation or surface roughness. It is assumed that *μ* and *A* depend on the parameter *ψ*, as follows: *μ* = *μ* (*ψ*) and *A* = *A* (*ψ*). By varying parameter *ψ* in inequality (2), we end up with:

(3) $$\frac{\partial }{2\partial t}\left(\delta^{2}S\right)=\frac{{\left(WV\right)}^{2}}{\lambda {\left(TA\right)}^{2}}\left[{\left(\frac{\partial \mu }{\partial \psi }\right)}^{2}A-\frac{\partial \mu }{\partial \psi }\frac{\partial A}{\partial \psi }\;\mu \right]\;{\left(\delta \psi \right)}^{2}\geq 0.$$

Self-organization can occur if inequality (3) is not fulfilled, i.e., the second term in the square brackets is positive. There are only four cases with the positive second term:

1. Case 1: If derivative ∂A/∂*ψ* is positive (*ψ* increases, *A* increases), ∂*μ*/∂*ψ* must be positive (as *ψ* increases *μ* must increase).
2. Case 2: If derivative ∂*A*/∂*ψ* is positive (*ψ* decreases, *A* decreases), ∂*μ*/∂*ψ* must be positive (as *ψ* decreases *μ* must decrease).
3. Case 3: If derivative ∂*A*/∂*ψ* is negative (*ψ* decreases, *A* increases), ∂*μ*/∂*ψ* must be negative (as *ψ* decreases *μ* must increase).
4. Case 4: If derivative ∂*A*/∂*ψ* is negative (*ψ* increases, *A* decreases), ∂*μ*/∂*ψ* must be negative (as *ψ* increases *μ* must decrease).

Cases 1 and 3 correspond to a situation when the real contact area can infinitely increase up to the instant when counterbodies stick together. Thus, a seizure can be considered as an effect that reduces wear (unconstructive self-organization) [11,14]. In practice, it is not always observed that counterbodies tend to stop moving. The cohesive bonding between the atoms can be broken by induced stress, therefore adhesive wear occurs and a morphology of contact surfaces changes instantly. In other words, an indication of adhesive wear regime in the test might be the COF increase. On the other hand, Cases 2 and 4 correspond to the situation when the real contact area can decrease up to a fictional loss of contact between sliding counterbodies, resulting in COF and wear decrease (constructive self-organization). At a certain instant, the pressure on certain contact points strongly increases, causing material collapse. The harder material can withstand higher stress than softer one, therefore Cases 2 and 4 can correspond to the abrasive wear. In analogy with the adhesive wear, the COF decrease can be associated with the abrasive wear.

The application of obtained inequality (3) to the analysis of the experimental data can be hampered due to the estimation of the behavior of *ψ*, *μ* and *A* parameters, as they can vary during the different stages of sliding.

## 4. Results and Discussions

**Figure 1:**
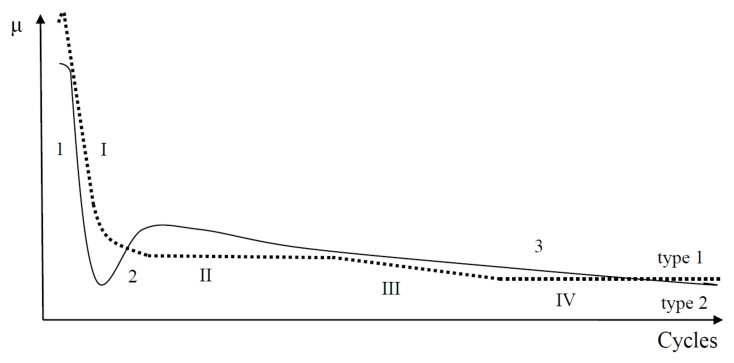
Schematics of two types of coefficient of friction (COF) versus cycles curves observed during reciprocation sliding wear test on diamond films.

Two typical COF versus cycles curves (with some variations) were observed in sliding tests with NCD [26,31,33,38] and MCD [34,35] films (Figure 1). The shape and length of stages 1–3 and I–IV can vary for the tests that are performed on the same sample under the same test conditions. Stage IV was observed in the COF curve of type 1, which corresponds to the steady state regime of sliding. The COF curve of type 2 corresponds to the running-in regime of sliding.

**Figure 2:**
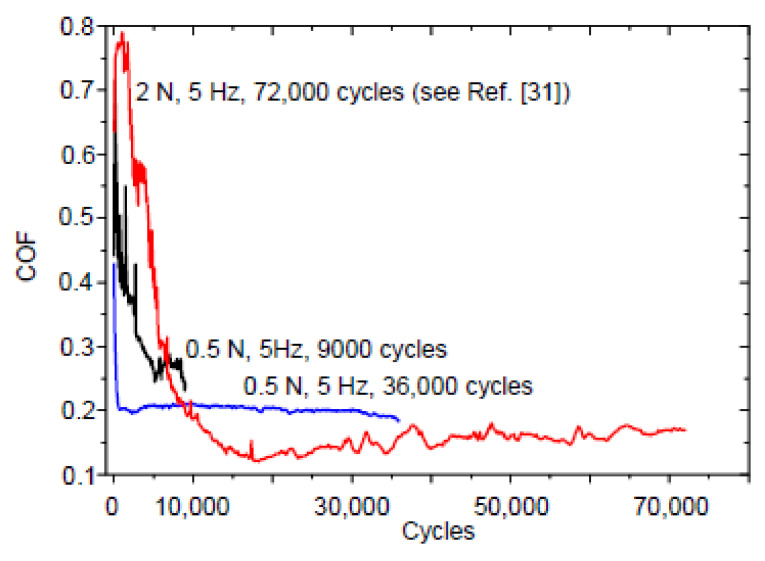
COF versus cycles curves taken on the 4.8 μm thick nanocrystalline diamond (NCD)-1 film.

**Figure 3:**
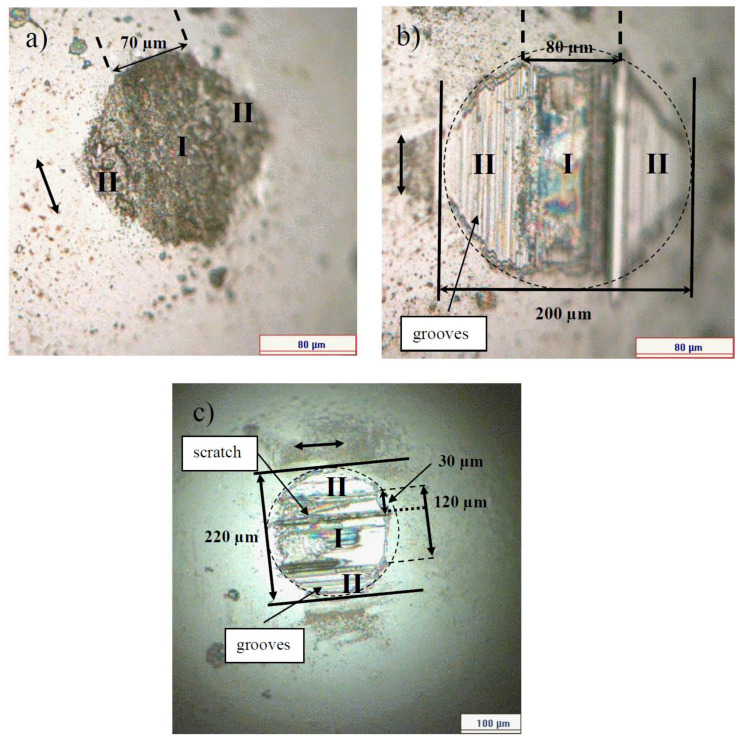
Surface morphology of the Si3N4 balls after the sliding wear tests on the 4.8 μm thick NCD-1 film. The test parameters were as follows: (**a**) 0.5 N, 5 Hz, 9000 cycles; (**b**) 0.5 N, 5 Hz, 36,000 cycles; and, (**c**) 2 N, 5 Hz, 72,000 cycles.

Figure 2 shows the COF versus cycles curves taken on the 4.8 μm thick NCD-1 film. The shape of the curve after the test with 72,000 cycles is similar to the shape of type 2 curve (Figure 1). The surface morphology of the Si_3_N_4_ balls after the tests with 9000 and 36,000 cycles (0.5 N, 5 Hz) and 72,000 cycles (2 N, 5 Hz) is shown in Figure 3. Circles (dashed lines) are shown as a guide for eyes.

**Figure 4:**
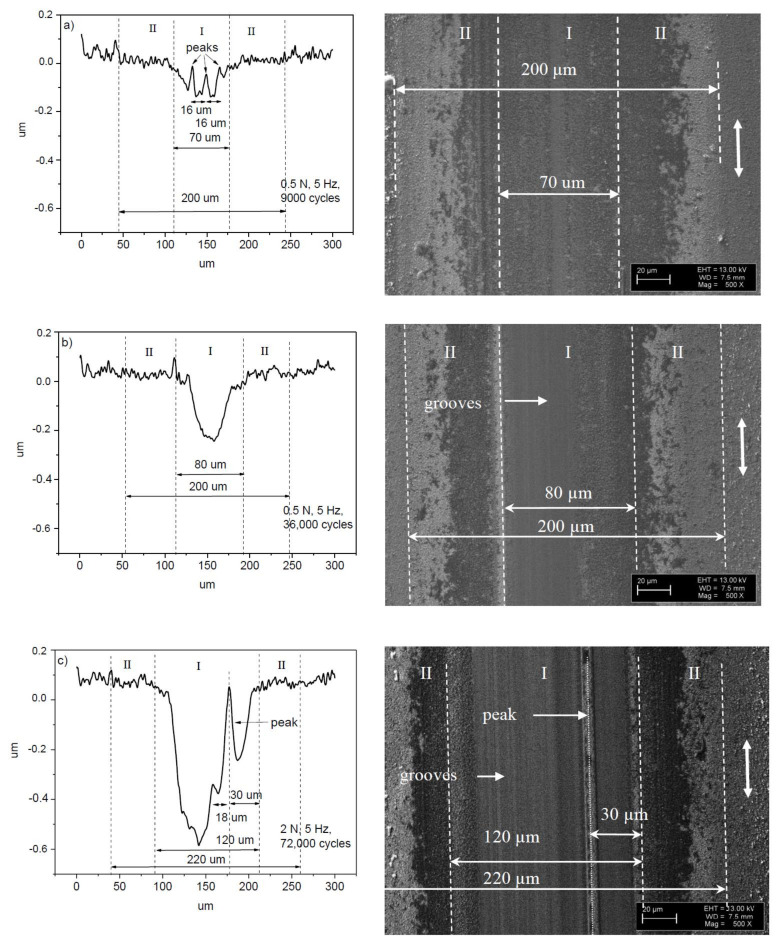
Line scans (see Ref. [31]) and SEM images taken on the wear scars of the 4.8 μm thick NCD-1 film. The sliding wear test parameters were as follows: (**a**) 0.5 N, 5 Hz, 9000 cycles; (**b**) 0.5 N, 5 Hz, 36,000 cycles; and, (**c**) 2 N, 5 Hz, 72,000 cycles.

The surface of the ball after 9000 cycles is considerably smoother than that after 36,000 cycles, namely no grooves and scratches were observed. A stripe-like area and two semicircle-like areas are marked by I and II on the ball surface, respectively. The morphological patterns on the ball surface for both of the areas look the same (Figure 3a). However, the patterns within the areas I and II look differently after the test with 36,000 cycles (Figure 3b), indicating the dissimilarity of wear regimes at the different parts of the ball. This can mean that real contact areas between the ball and film within the areas I and II are different, due to the difference in surface morphology. In other words, single type of wear regime dominates for the test with 9000 cycles of sliding, and for the longer tests (36,000 cycles), a differentiation of wear mechanisms occurs, namely at least two types of wear regimes can be distinguished. The width of the area I increases steadily from 70 to 120 μm (Figure 3 and Figure 4).

**Figure 5:**
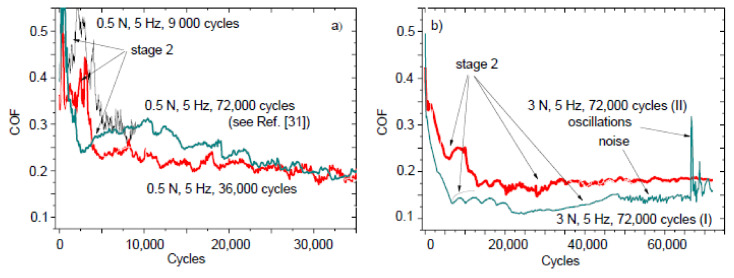
COF versus cycles curves taken on the 22 μm thick NCD-1 film: (**a**) at the 0.5 N; (**b**) 3 N normal load condition. Two tests at the 3 N normal load were taken at the different places on the sample.

Figure 5 shows the COF versus cycles curves that were taken on the 22 μm thick NCD-1 film. The film roughness S_q_ (root mean square) was 99 nm in comparison with 62 nm for 4.8 μm thick film [31]. The COF curves that are similar to the type 2 curve (Figure 1) can be seen for the tests that were taken at the 0.5 N load (Figure 5a). The position and shape of the stage 2 for the tests with 9000 and 36,000 cycles differ from the one with 72,000 cycles. It indicates that the running-in regime of sliding can vary for similar tests, due to probably fluctuation of the surface roughness. Variation of tribological behavior in sliding tests with the higher load (3 N, 5 Hz, 72,000 cycles) was found as well (Figure 5b).

**Figure 6:**
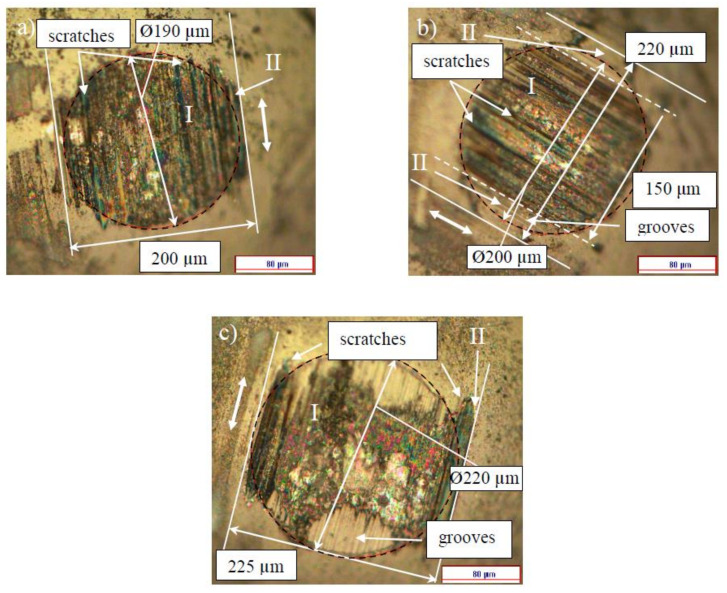
Surface morphology of the Si3N4 balls after the sliding wear tests on the 22 μm thick NCD-1 film. The sliding wear test parameters were as follows: 0.5 N, 5 Hz, 9000 cycles (**a**), 0.5 N, 5 Hz, 36,000 cycles (**b**), and 3 N, 5 Hz, 72,000 cycles (I) (**c**).

**Figure 7:**
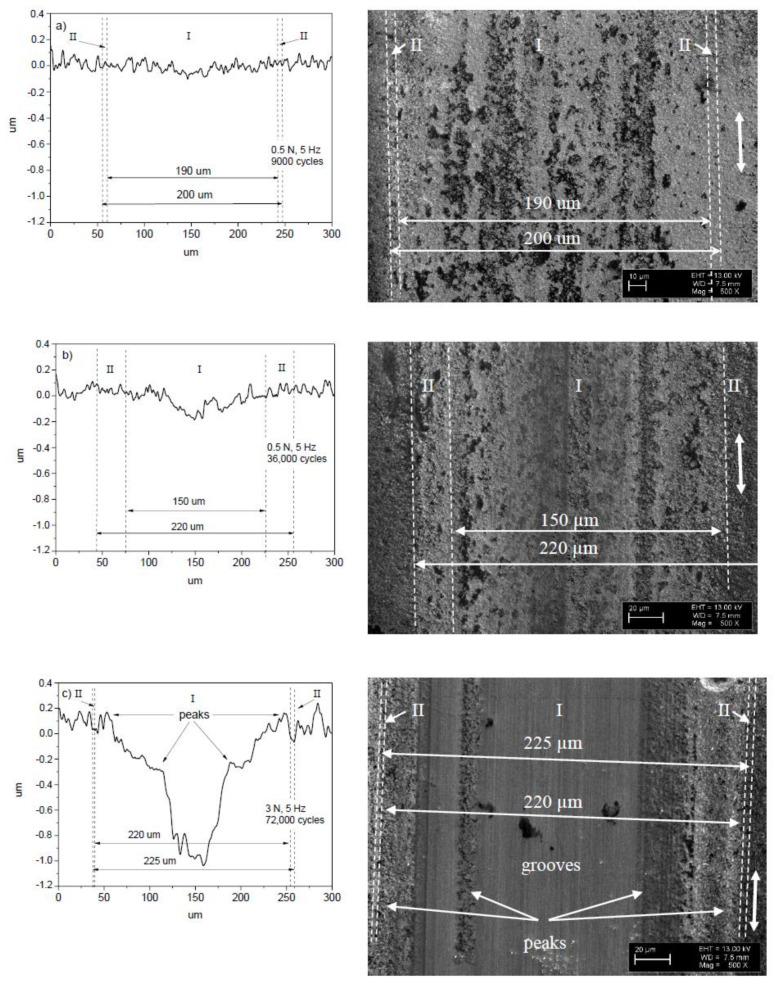
Line scans (see Ref. [31]) and SEM images taken on the wear scars of the 22 μm thick NCD-1 film. The sliding wear test parameters were as follows: (**a**) 0.5 N, 5 Hz, 9000 cycles; (**b**) 0.5 N, 5 Hz, 36,000 cycles; and, (**c**) 3 N, 5 Hz, 72,000 cycles (I).

Figure 6 shows the surface morphology of the balls after the sliding tests on the 22 μm thick NCD-1 film. There is some difficulty in distinguishing between areas I and II on the wear scar after the test with 9000 cycles, however, these areas can be clearly discriminated after 36,000 cycles (Figure 6b). The shape of the wear scars after 9000 cycles is circle-like in contrast to ones that were observed on thinner film (Figure 3a), likely due to a weaker deformation of the thicker film [31]. In addition, the increase of area II suggests that the film deformation (deflection) increases gradually for the tests from 9000 to 36,000 cycles (Figure 6a,b). This finding indicates fatigue [31]. Interesting that the width of the wear scars on the balls (Figure 3 and Figure 6) after the shorter (9000 cycles) (not shown in Figure 3a) and longer (36,000 and 72,000 cycles) tests is nearly the same, i.e., about 200–225 μm, and is nearly independent of film thickness, load (0.5, 2 and 3 N), and duration. It worth comparing the size of areas I and II for both of the films (4.8 and 22 μm) that were tested in similar tests, i.e., at 0.5 N and 5 Hz (36,000 cycles) (Figure 3b, Figure 4b, Figure 6b and Figure 7b). The size of area I is 80 and 150 μm, and area II is 120 and 70 μm for 4.8 and 22 μm thick films, respectively. The difference in size of areas as well as in the shape and depth of line scans (Figure 4b and Figure 7b) clearly indicate the difference in wear regimes. In addition, morphological patterns after the tests on 22 μm thick film differ from those on the 4.8 μm thick film. Scratches can be observed already after 9000 cycles of sliding, which is probably due to the higher roughness of the 22 μm thick film.

The position of the scratch on the ball surface corresponds to the position of the peak on the wear scar (Figure 3c and Figure 4c). The strongest wear of the ball occurs within the scratch, and, correspondingly, the lowest wear of the diamond film is on the peak. For the longer tests, a highly asymmetric profile of the wear scars was often observed on the different types of diamond films [31,34,35]. In other words, wear can be locally highly non-uniform. Thus, a variation of the real contact area can be expected within the contact zone. For instance, a contact between two bodies could take place mainly between the peak and area II on the film, and between the scratch and area II on the ball, thus a reduction in real contact area can be expected within the area I (Figure 3c and Figure 4c). Another type of the contact between the ball and film can be distinguished after the observation of the surface morphology of the ball and film in Figure 6c and Figure 7c. There is a correspondence between the positions of the scratches on the ball and the peaks on the film, which indicates the primary regions of contact between the ball and film. Thus, the reduction in the real contact area can be expected within the central part of the wear scar (area I). In conclusion, grooves, scratches, and peaks of the different shapes and sizes were observed on the surface of balls and films (Figure 3, Figure 4, Figure 6 and Figure 7), thus a diverse range of contact types between the film and ball can be expected, leading to the different tribological behavior.

**Figure 8:**
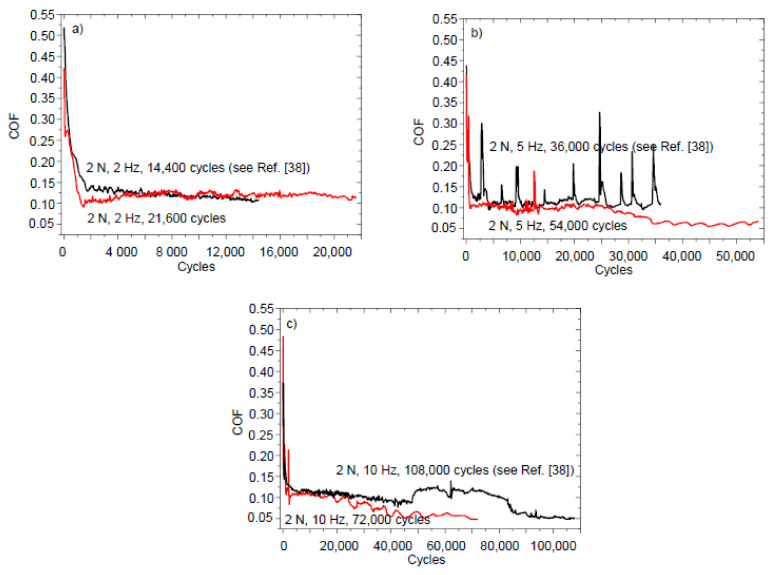
COF versus cycles curves taken on the NCD-2 film. The sliding wear test parameters were as follows: (**a**) 2 N and 2 Hz; (**b**) 2 N and 5 Hz; and, (**c**) 2 N and 10 Hz.

Figure 8 shows the COF versus cycles curves taken on the 0.8 μm thick NCD-2 film. Generally, the shape of the curves in Figure 8 is similar to the shape of the curve type 1 (Figure 1). Type 2 curve was observed, for instance, for the test with 21,600 cycles (2 N, 2 Hz) (Figure 8a). Stage II of the curve type 1 corresponds to the sliding regime with a nearly constant or slightly descending COF value. The duration of this stage varies, i.e., it decreases with an increasing of sliding velocity. The COF value decreases for the longer tests and stabilizes to value 0.05 at stage IV.

**Figure 9:**
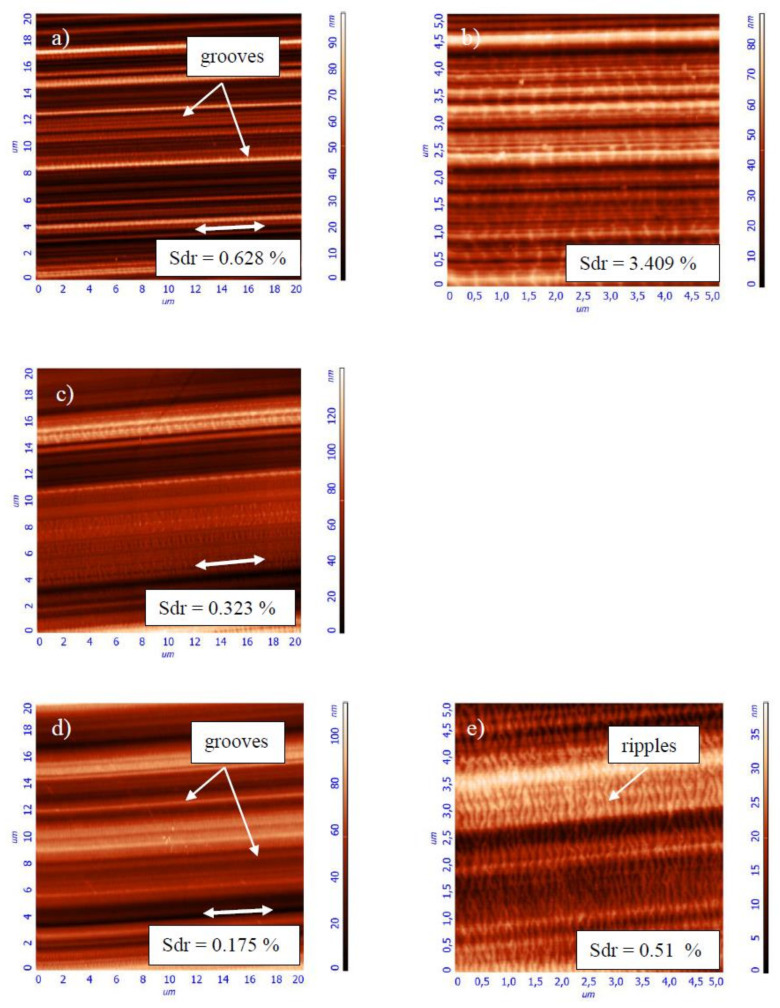

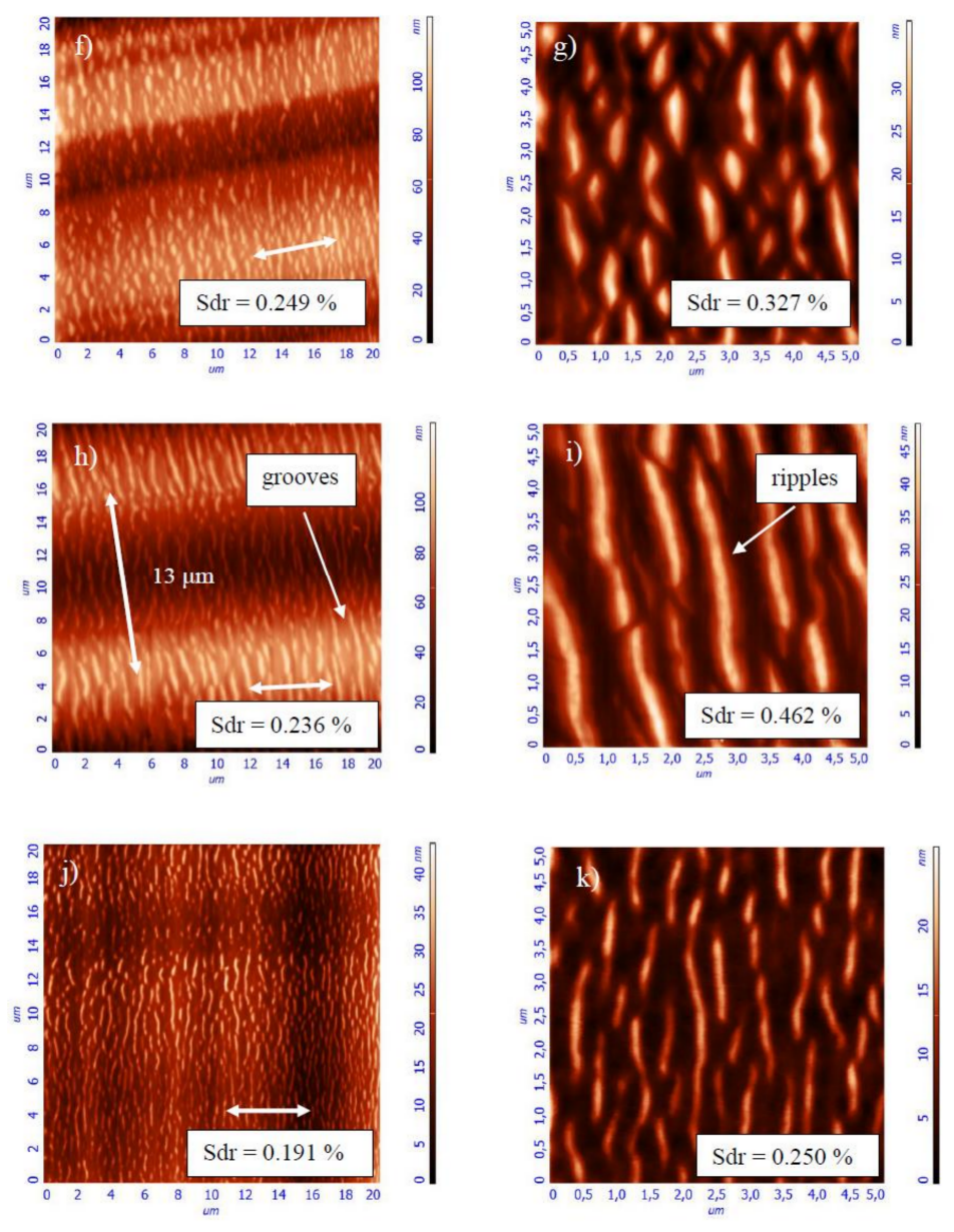
Atomic force microscopy (AFM) images (see Ref. [38]) taken on the wear scars of the NCD-2 film after the sliding wear tests. The test parameters were as follows: (**a**,**b**) 2 N, 2 Hz, 14,400 cycles; (**c**) 2 N, 2 Hz, 21,600 cycles; (**d**,**e**) 2 N, 5 Hz, 36,000 cycles; (**f**,**g**) 2 N, 5 Hz, 54,000 cycles; (**h**,**i**) 2 N, 10 Hz, 72,000 cycles; and, (**j**,**k**) 2 N, 10 Hz, 108,000 cycles.

Figure 9 shows the surface patterns that were observed on the NCD-2 film after sliding tests. AFM images were taken at the central part of corresponding wear scars. The real contact area between the sliding counterparts can be characterized by the surface area ratio parameter Sdr, see Experimental. The Sdr value is greater for shorter tests (Figure 9), i.e., for the tests with 2 Hz, and it is smaller for the longer tests with 5 and 10 Hz, which can be clearly seen in the higher resolution images. Higher contact surface area for shorter tests means more adhesive contact between the counterbodies. For the longer tests, the Sdr value decreases together with a COF value decrease and stabilization. In other words, the decrease of contact area within the central part of wear scars for the longer tests is similar to the that were results obtained on NCD-1 film, see discussion above.

More similarity between the surface morphology of NCD-1, NCD-2, and NCD-3 films can be found in Figure 3, Figure 4, Figure 6 and Figure 9. The distance between small peaks within the wear scar (Figure 4a) is about 16 μm for the NCD-1 film, which is in good agreement with the distance between the peaks that were found on the NCD-2 film, i.e., 13 μm (Figure 9h). The height of peaks for both of the patterns is similar, namely about 100 nm, see height bar in Figure 9h. In addition, the distance between two peaks is 18 μm (Figure 4c), which could indicate that the large peak (Figure 4c) is formed from a smaller peak, which is similar to ones in Figure 4a. On the other hand, the emergence of shallow grooves and deeper scratches on the surface of balls (Figure 3 and Figure 6) also suggests the dynamic formation and annihilation of these peculiarities during sliding. The groove and ripple patterns on the surface of wear scars were observed on all NCD (NCD-1, NCD-2, and NCD-3) films, indicating the formation of the well-ordered spatial structures during sliding [11,40].

The tribological behavior of MCD films was investigated in our studies [34,35]. The shape of the COF versus cycles curves (not shown) corresponds to the type 2 curve (Figure 1). The grooves, ripples and film deflection were observed [34,35]. The wear scar profile (not shown) on the film similar to one shown in Figure 4c (i.e., with the large peak within the wear scar) was found after the sliding test on the MCD film [34].

There is a correlation between Cases (1–4) describing conditions for self-organization to occur, the shape of the COF curves (Figure 1, Figure 2, Figure 5 and Figure 8) and the morphological patterns on the balls (Figure 3 and Figure 6) and films (Figure 4, Figure 7 and Figure 9) surfaces. An interpretation of results needs an estimation of influence of all types of friction and wear mechanisms to tribological behavior. The principal mechanism (or mechanisms) is mostly predetermined the type of COF curve, for instance, the emergence of either stage 2 or II. The shape of the COF versus cycles curves can depend on many processes [41,42]. Both kinds of stages (2 and II) were found in the COF curves after the tests on the same film tested under the same test conditions (Figure 8a). A more complete description of the friction needs to take into account the possible synergetic effects or the mutual relationships between the all involved processed as well.

At the beginning of sliding test, the surface asperities interlocking results in material fracture and self-polishing [21,22,23]. These wear mechanisms, as well as passivation of dangling bonds and bonds breaking, formation of carbonaceous layer, and surface deformation influence on the diamond film tribological behavior at the early stages of the sliding, see stages 1 and I (Figure 1). The real contact area increases due to the initial polishing of surface asperities. In the case of surface roughness alteration, parameter *ψ* decreases (as the roughness decreases) along with the decrease of *μ* and increase of *A*. This situation cannot correspond to any of the mentioned above cases, see Theoretical background. Therefore, the fracture and initial polishing mechanisms cannot be related with self-organization, which is in good agreement with conclusions by Gershman et al. [14]. In the case of surface deformation, *ψ* increases as the film deflection increases [31], together with a decreasing in *μ* and an increasing in *A*, thus no self-organization occurs as well. The decrease of COF value at stages 1 and I can be related with the formation of carbonaceous layer as well, however during some initial period of sliding, the fracture and the initial polishing mechanisms should dominate, as formation of the carbonaceous layer needs a certain period of time [26].

An indication of self-organization can be a change of the wear rate [14]. The evaluation of the wear rate of the tribological system with deflection is a challenge as the surface deformation influences on the estimation of the wear volume [31,32]. The change of the wear rate can be related to a change of the wear regime. For instance, after relatively similar stages 1 and I, a bifurcation takes place, and the COF value follows the dynamics corresponding to either stage 2 or II (Figure 1). Different processes (dangling carbon bonds passivation and bonds breaking, adhesive and abrasive wear, film deformation, and formation of carbonaceous layer, etc.) can be distinguished for the later stages of sliding, which can cause to formation of the dissipative structures. A fraction of the friction energy can be dissipated into the breaking or passivation of the dangling bonds, formation of carbonaceous layer, and well-ordered morphological patterns, etc. [14]. A thermodynamical analysis of mentioned processes should show if they proceed with negative or positive entropy production, thus decreasing or increasing wear, respectively.

Tribological behavior during stage 2 (Figure 1) can be explained by using inequality (3), as follows. With regard to the role of film deformation, the film deflection increases at the early stages of sliding [31], i.e., parameter *ψ* increases. The COF value increases, i.e., d*μ*/d*ψ* > 0. The deformation of film causes the real contact area increase, thus ∂*A*/∂*ψ* > 0 as well. Therefore, the behavior corresponds to the Case 1 and adhesive wear regime of sliding. A correlation between the surface corrugation and COF behavior of NCD-1 film can be found in Figure 5b, Figure 6c and Figure 7c. Noise, oscillations, and an increase of COF value can be observed in Figure 5b, likely indicating a seizure-like contact between the ball and film. Increasing contact surface area between the peaks and corresponding scratches (Figure 6c and Figure 7c) due to the shape of these peculiarities could cause to adhesive wear and seizure. In other words, the surface corrugation increases at stage 2, i.e., *ψ* increases along with *μ* and A. Therefore, it corresponds to the Case 1 as well. The formation of the groove patterns on the early stages of sliding (Figure 9) can be additional evidence of an adhesive wear regime. For the NCD-2 film, the denser arrays of grooves and the highest Sdr value were observed after shorter tests (Figure 9a,b). The predominance, or at least the manifestation, of the adhesive wear regime can be expected for these tests due to a higher contact area between counterbodies. The existence of the adhesive wear regime at the early stages of sliding (parts 2 and II in Figure 1) can be easily understood as a consequence of the wear mechanisms occurring within parts 1 and I (Figure 1), namely due to the initial polishing of the asperities the contact area increases, see discussion above. However, further increase of contact area can result in COF value increase (part 2). In the case of type 1 curve (Figure 1 and Figure 8), the nearly constant COF value for part II indicates an interplay between adhesive and other friction and wear mechanisms. It was assumed that the real surface area can infinite increase in the case of adhesive wear, see Cases 1 and 3 in Theoretical background. A fractal surface is an object with the infinite surface area, see, for example, Koch fractal [1].

The justification of the type of wear regime occurring during stages II, 3, III, and IV of the COF curves is as follows. First, the analysis of tribological behavior of diamond films for stages 3 and III is presented. The COF value decreases at stages 3 and III. The wear mechanisms can differ within the separate regions of contact zone. The real contact area can locally decrease, for instance, within the central part of the wear scar, as it was shown for NCD-1 and NCD-2 films, see discussion above. In the case of NCD-2 film, the surface morphology alteration leads to the formation of larger grooves, and finally to relatively flat contact surface within the central part of the wear scar (Figure 9f–k). It can be a leading factor, which influences tribological behavior. The decrease of both *A* and COF corresponds to the Cases 2 and 4. It is interesting that variations of *ψ* is not important, i.e., independent of changes in surface roughness and film deformation constructive self-organization can occur. In conclusion, the dominant type of wear mechanism corresponding to stages 3 and III can be the abrasive wear. The relationship between the bonds passivation and bonds breaking, adhesive and abrasive wear, film bending and formation of carbonaceous lubricating layer can lead to the COF value equalization observed for the stages II and IV. The COF value slightly varies during the steady stage regime IV (Figure 8), which can be attributed to the interplay between different mechanisms. However, the intensity of COF value fluctuations is lower in comparison to the processes occurring at the earlier stages of sliding (I–III and 1–3).

The surface patterns on the balls and films appear as a consequence of a dynamic coexistence of ripples, grooves, scratches, and peaks. The grooves can undergo a structural transformation into the scratches and the peaks can be destroyed, which changes the landscape of the contact zone on the ball and film. A close relationship between adhesive and abrasive wear explained in terms of permanently changed surface morphology is in good agreement with the results of a study by Mortazavi and Nosonovsky [43]. In this study, the running-in period of sliding was explained as the adaptive self-organization process or the mutual adjustment of counterbodies.

## 5. Conclusions

The friction and wear of diamond films originate from the complex processes. There is a relationship between the fracture and the initial polishing of the surface asperities, dangling bonds passivation and bonds breaking, formation of carbonaceous layer, adhesive and abrasive wear, and film deformation during the different stages of sliding. The adhesive and abrasive wear of diamond films can be explained as a consequence of self-organization in the tribological system. After an initial period, the bifurcation behavior is caused by instabilities that were induced by surface roughness alteration, film bending, etc. The bifurcation denotes a sudden change of the real contact area. The increase of the real contact area between the sliding counterparts indicates the adhesive wear regime. On the other hand, the decrease of the real contact area corresponds to the abrasive wear.
